# Clinical significance of Caveolin-1, Caveolin-2 and HER2/neu mRNA expression in human breast cancer

**DOI:** 10.1038/sj.bjc.6602029

**Published:** 2004-08-10

**Authors:** Y Sagara, K Mimori, K Yoshinaga, F Tanaka, K Nishida, S Ohno, H Inoue, M Mori

**Affiliations:** 1 Department of Breast Oncology, National Kyushu Cancer Center, 3-1-1 Notame, Minami-Ku, Fukuoka 811-1395, Japan; 2 Department of Surgery, Medical Institute of Bioregulation, Kyushu University, 4546 Tsurumihara, Beppu 874-0838, Japan

**Keywords:** caveolin, HER2/neu, breast cancer, trastuzumab, tumour size, hormonal receptor

## Abstract

Caveolin-1 and -2 (CAV1, CAV2) are closely linked genes localised to the fragile region of 7q31 (FRA7G), and loss of heterozygosity involving this region has been reported in breast cancer. Several studies have suggested that CAV1 is a negative regulator of HER2/neu signal transduction *in vitro*. However, the clinical significance of CAV1 in breast cancer has not yet been clarified. We examined quantitatively the mRNA levels of CAV1, CAV2 and HER2/neu in 162 cases of breast cancer using real-time PCR. Caveolin-1 and -2 protein expression was also examined by Western blotting and immunohistochemistry. We then evaluated for correlations between CAV1, CAV2 and HER2/neu gene expression and clinicopathologic factors in the 162 breast cancer cases. Results showed higher HER2/neu mRMA levels and lower CAV1 and CAV2 mRMA levels in breast cancer tissues than in corresponding normal tissues (*P*<0.001). Caveolin-1 and -2 protein expression levels were also suppressed in cancer tissues compared to normal tissues by Western blotting. Immunohistochemistry revealed that CAV1 and CAV2 proteins were abundantly expressed in mammary gland myoepithelial cells, but only weakly in ductalepithelial cells. Reduced CAV1 mRNA level was significantly associated with increasing tumour size (*P*=0.041), and negative oestrogen receptor status (*P*=0.021). There was also a significant association between low CAV2 mRNA level and negative progesterone receptor status (*P*=0.013), and between high HER2/neu mRNA level and negative hormonal receptor status (ER, *P*=0.029, PgR, *P*=0.019). While there was no relationship between HER2/neu and CAV1 mRNA levels, a significant association between CAV1 and CAV2 mRNA levels was observed (*P*<0.001). Our results indicated that CAV1 suppression correlated closely with that of CAV2 in breast cancer, that CAV1 level was inversely correlated with tumour size, and that CAV1 and CAV2 levels were correlated with hormonal receptor status. Therefore, CAV1 and CAV2 play an important role in tumour progression in breast cancer patients.

Caveolae mediate molecular transport, cell adhesion and signal transduction activities in the cell (Couet *et al*, 1997; Zhang *et al*, 2000; Razani *et al*, 2001). Caveolin-1 (CAV1), the major coat protein of caveolae, has been reported to interact with various intracellular signalling molecules including growth factors such as epidermal growth factor receptor (EGFR) (Couet *et al*, 1997; Engelman *et al*, 1998; Kim *et al*, 2000) and oestrogen receptor (ER) (Razandi *et al*, 2002; Zschocke *et al*, 2002). Some studies have suggested that caveolin may function as a negative regulators of signal transduction to HER2/neu, member of the EGF family. In recent years, trastuzumab (Herceptin®) has been used as the first-line agent in the treatment of recurrent breast cancers that overexpress HER2/neu. Interestingly, some breast cancer patients with HER2/neu-overexpressing tumours do not respond well to trastzumab treatment.

Several lines of evidence have suggested that caveolin expression in malignancies may be clinically significant. For example, CAV1 expression is increased in prostate cancer (Yang *et al*, 1999), oesophageal cancer (Kato *et al*, 2002) and ovarian cancer (Davidson *et al*, 2001), but reduced in colon cancer (Bender *et al*, 2000), lung cancer (Ho *et al*, 2002) and sarcoma (Wiechen *et al*, 2001).

In the present study, we investigated the mRNA levels of CAV1, Caveolin-2 (CAV2) and HER2/neu in breast cancer tissues from 162 cases by the real-time PCR to evaluate the clinical significance of these genes.

## MATERIALS AND METHODS

### Surgical specimens

A totalof 162 female cases of breast cancer were available for study and included 93 cases with both tumour and corresponding normal specimens, and 69 cases with only tumour specimens. Surgery was performed from October 1993 to October 1999 at the Medical Institute of Bioregulation, Kyushu University Beppu, and Oita Prefectural Hospital, Oita, Japan. Experimental protocols were authorised by a Bioethics Committee and informed consent was obtained from all patients in the study. The mean patient age was 55 years (range, 27–84 years), and median postoperative follow-up was 41 months (range, 1–96 months). Hormonal status was identified in 125 cases. All tumours are histopathologically diagnosed as breast cancer. To avoid degeneration, cancer tissue centres and corresponding cancer-free tissues were snap frozen in liquid nitrogen immediately after excision, handled carefully to avoid contamination with RNase, and stored at −80°C until use. mRNA quality was assessed in several representative cases using a Bioanalyzer (Agilent Technologies, Japan) to exclude unsuitable samples. Results showed samples were appropriate for further study.

### Cell culture

Human breast cancer cell lines, MCF-7, YMB-1, SKBR-3, MRKnu-1 and CRL-1500 were obtained from the Cell Resource Center at the Biochemical Research Institute of Development, Aging and Cancer (Tohoku University, Sendai, Japan). YMB-1 was maintained in DMEM and MCF-7, SKBR-3, MRKnu-1 and CRL-1500 were maintained in RPMI 1640. All cells lines were supplemented with 10% FBS and incubated at 37°C in a 5% humidified CO_2_ atmosphere.

### Preparation of cDNA from tissue specimens

Total RNA was extracted using the acid guanidinium thiocyanate/phenol/chloroform extraction (AGPC) method. All samples were treated with DEPC in Eppendorf tubes (Eppendorf, Germany) and handled with gloves to avoid RNase contamination. Total RNA aliquots were reverse transcribed into cDNA using oligo-dT primers.

### Quantitative analysis of mRNA of CAV1, 2 and HER2/neu

Reverse transcriptase reactions were performed as previously described (Mori *et al*, 1993). Real-time PCR was performed using the iCycler iQ detection system (Bio-Rad, Tokyo, Japan) and iQ SYBR Green Supermix. Reactions were performed in 96-well plates with Optical-Quality 8-tube Strips (Bio-Rad). The primer sequences for HER2/neu, CAV1, 2 and glyceraldehyde-3-phosphate-dehydrogenase (GAPDH) are detailed in Table 1

**Table 1:** Primers sets for the amplification of *HER2/neu, CAVEOLIN 1, CAVEOLIN 2 and glycelaldehyde-3-phosphate dehydrogenase*

**Gene**	**Primer**
*HER2/neu*	Upper: 5′ CCCCCAAAGCCAACAAAGAAA 3′
	Lower: 5′GCCGCACATCCTCCAGGTAGC 3′
*Caveolin-1 (CAV1)*^a^	Upper: 5′ AAGGGACACACAGTTTTGACG 3′
	Lower: 5′ TTGGCACCAGGAAAATTAAAA 3′
*Caveolin-2 (CAV2)*	Upper: 5′ GGCGGACGTACAGCTCTTCAT 3′
	Lower: 5′ GCCAGGAACACCGTCAGGAAC 3′
*GAPDH*	Upper: 5′ GTCAACGGATTTGGTCTGTATT 3′
	Lower: 5′ AGTCTTCTGGGTGGCAGTGAT 3′

a It is designed so that the arrangement of mutation of Caveolin-l (reported by Cancer Research 61, 2361–2364, March 15, and 2001) might be included.

. The reactions for both of them were subjected to 40 cycles for 30 s at 95°C, 1 min at 60°C, and 1 90 s at 72°C. Increases in fluorescence were measured in real time during the extension step.

### Fluorescence *in situ* hybridisation (FISH) of HER2/neu

Fluorescence *in situ* hybridisation on breast cancer cell lines was performed at Ohtsuka Assay Inc., according to the PathVysion (Vysis, Inc., Illinois, USA) protocol. Briefly, cultured breast cancer cell lines on microscope slides were hydrated with Hemo-De clearing agent (Vysis, Inc.) and a graded alcohols series. Slides were air dried, pretreated (80°C, 30 min), and digested with protease (37°C, 10–20 min) before hybridising with fluorescent-labelled probes for the HER-2/*neu* gene and chromosome 17 alpha-satellite DNA. Probes were premixed and predenatured in hybridisation buffer for ease of use. Nuclei were counterstained with intercalating fluorescent counterstain 4′-6′-diamidino-2′-phenylindole (DAPI). HER2 amplification ratio was calculated as: HER2 total signal count/chromosome 17 total signal count. Presence of HER2 amplification was defined as an HER2 amplification ratio of greater than 2.00.

### Western blotting and immunohistochemistry for CAV1 and CAV2

For Western blot analysis, proteins were extracted from normal and tumour tissues in five representative breast cancer cases. Proteins were subjected to SDS–PAGE in 15% acrylamide gels under reducing conditions and transferred to Immobilon-P membranes (Millipore, Bedford, MA, USA). After blocking with 5% nonfat dry milk and 0.05% Tween-20 in PBS, blots were incubated with CAV1 (BD Biosciences, CA, USA) and CAV2 (Santa Cruz, CA, USA) mAb, used as culture supernatants diluted 1 : 2. After several washings, blots were incubated for 1 h with goat anti-mouse IgG (1 : 5000) coupled to horseradish peroxidase, washed extensively, and developed using a chemiluminescence Western blotting kit (ECL, Amersham, Buckinghamshire, UK).

For immunohistochemistry, surgically resected specimens from the same five cases of breast cancer tissues as used above and the corresponding normal tissues were stored at −20°C until use. Frozen sections (4 *μ*m) were put onto silicon-coated glass slide and stained with monoclonal antibodies, as above. Staining for CAV1 and CAV2 was performed on adjacent sections. Immunohistochemical staining was performed as previously described (Mori *et al*, 2000) using serum specific for human monoclonal CAV1 and 2 antibodies.

### Statistical analysis

**Figure 1:**
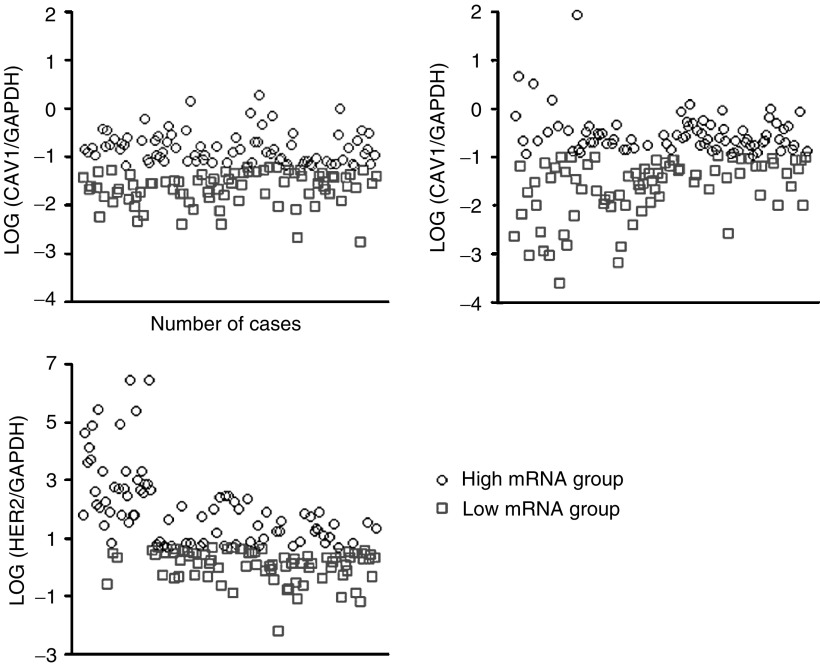
mRNA levels of CAV1, CAV2, and HER2 in breast cancer patients. We divided the 162 cases into 81 high and 81 low mRNA groups for each mRNA examined. LOG (CAV1 mRNA/GAPDH mRNA) varies between −2.75 and 0.27 (mean, −1.23), LOG (CAV2 mRNA/GAPDH mRNA) varies between −5.57 and 1.95 (mean, −1.13) and LOG (HER2 mRNA/GAPDH mRNA) varies between −2.18 and 6.43 (mean, 1.09).

mRNA levels of CAV1, CAV2 and HER2/neu were calculated with respect to GAPDH mRNA level. The 162 cases were diveided into high and low mRNA groups (with 81 in each group), according to mRNA level in each breast cancer (Figure 1

), and correlation between mRNA expression level and clinicopathologic factor were analysed. The predictive value of mRNA expression level for clinicopathological variables such as age, size of tumour, lymph node metastasis, lymph vessel invasion, vascular vessel invasion, oestrogen or progesterone receptor status were univariately tested using a *χ*^2^ test. Correlation of each mRNA level was analysed using the log rank test, and the cumulative survival rate was calculated by the Kaplan–Meier method. All analyses were performed using statistical software (Statview version 5.0; SAS Institute, Inc., Cary, NC, USA), with a *P*-value of less than 0.05 considered statistically significant.

## RESULTS

### Expression levels of CAV1, CAV2 and HER2/neu in normal breast tissue, breast cancer tissue and cell lines

**Figure 2:**
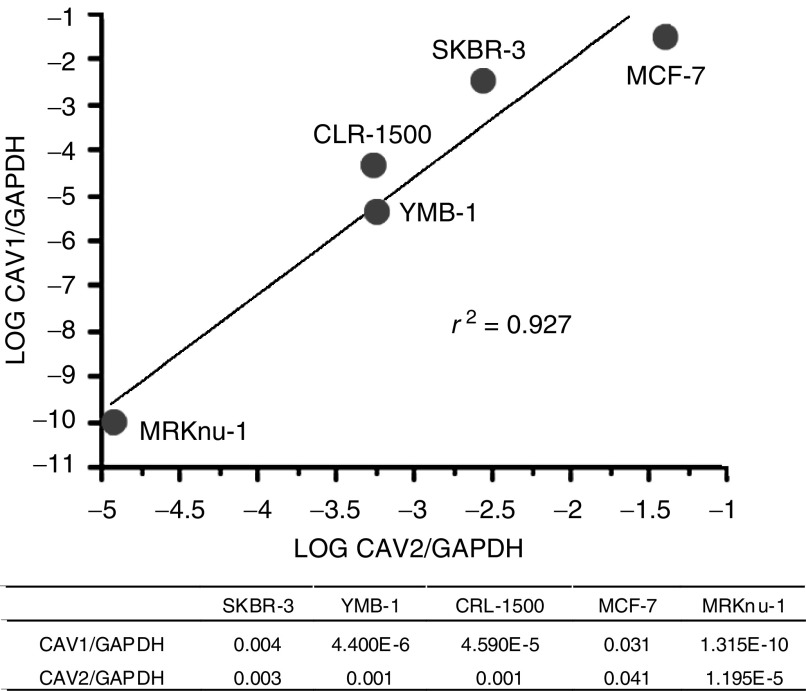
Expression of CAV1 and CAV2 mRNA in breast cancer cell lines. There was a statistical significant correlation between caveolin-1 (CAV1) and caveolin-2 (CAV2) expression in five breast cancer cell lines, adjusted by linear regression analysis. Caveolin-1 and CAV2 expression levels were adjusted according to GAPDH expression level. Letters A to E in the graph and table indicate the actual data for the five breast cancer cell lines.

**Figure 3:**
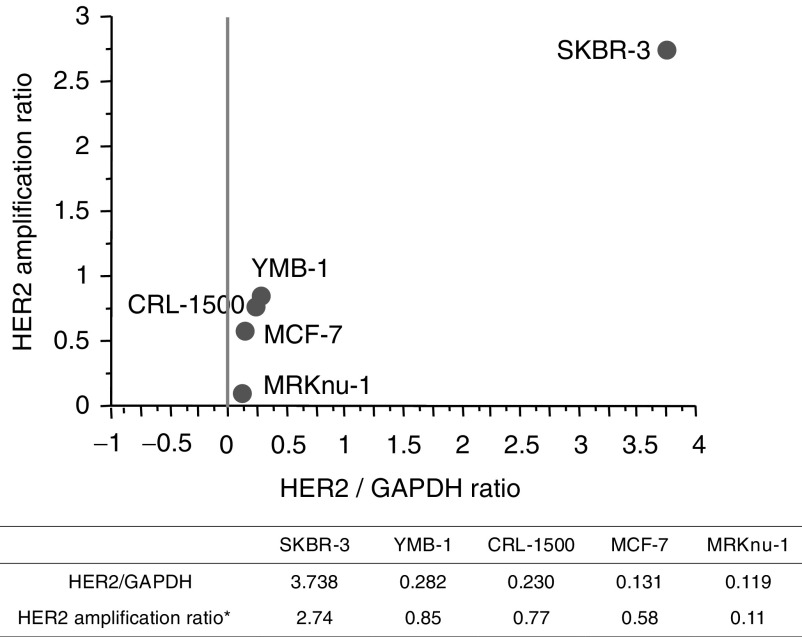
Expression of HER2/neu mRNA and HER2/neu amplification ratio in breast cancer cell lines. ^*^Fluorescence *in situ* hybridisation *HER2* amplification ratio=*HER2* total signal count/Chr 17 total signal count (*HER2* amplification was defined as *HER2* amplification ratio >2.00). There was a statistically significant correlation between HER2/neu expression and HER2/neu amplification (*R*=0.968, *R*=0.0068). The table shows actual data of *HER2/neu* expression and *HER2/neu* amplification.

**Figure 4:**
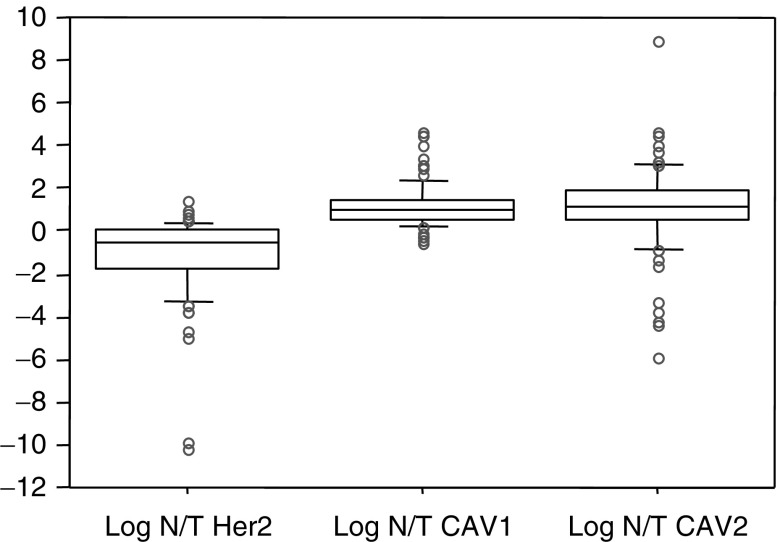
Comparison of CAV1, CAV2 and HER2/neu expression between breast cancer tissue (T) and the corresponding normal mammary gland (N). Average N/T ratio for CAV1, CAV2 and HER2/neu were 24.6±3.34, 37.9±4.42, and 1.15±0.33, respectively. Significantly higher CAV1 and CAV2 expression was observed in normal mammary glands, while HER2/neu was abundant in tumour tissues.

Low levels of CAV1 mRNA were detected in several breast cancer cell lines (Figure 2

), with CAV1 and CAV2 mRNA levels significantly correlated with each other (*r*^2^=0. 927). The SKBR-3 cell line exhibited the highest HER2/neu mRNA level of the breast cancer cell lines tested (SKBR-3, YMB-1, CRL-1500, MCF-7, MRKnu-1), and FISH detected HER2/neu overexpression only in SKBR-3 cells (Figure 3

). Thus, HER2/neu mRNA levels were concordant with HER2/neu FISH results. Comparison of breast cancer samples and corresponding normal breast tissues for 93 cases showed higher HER2/neu mRNA levels and lower CAV1 and CAV2 mRNA levels (*P*<0.001) (Figure 4

).

**Figure 5:**
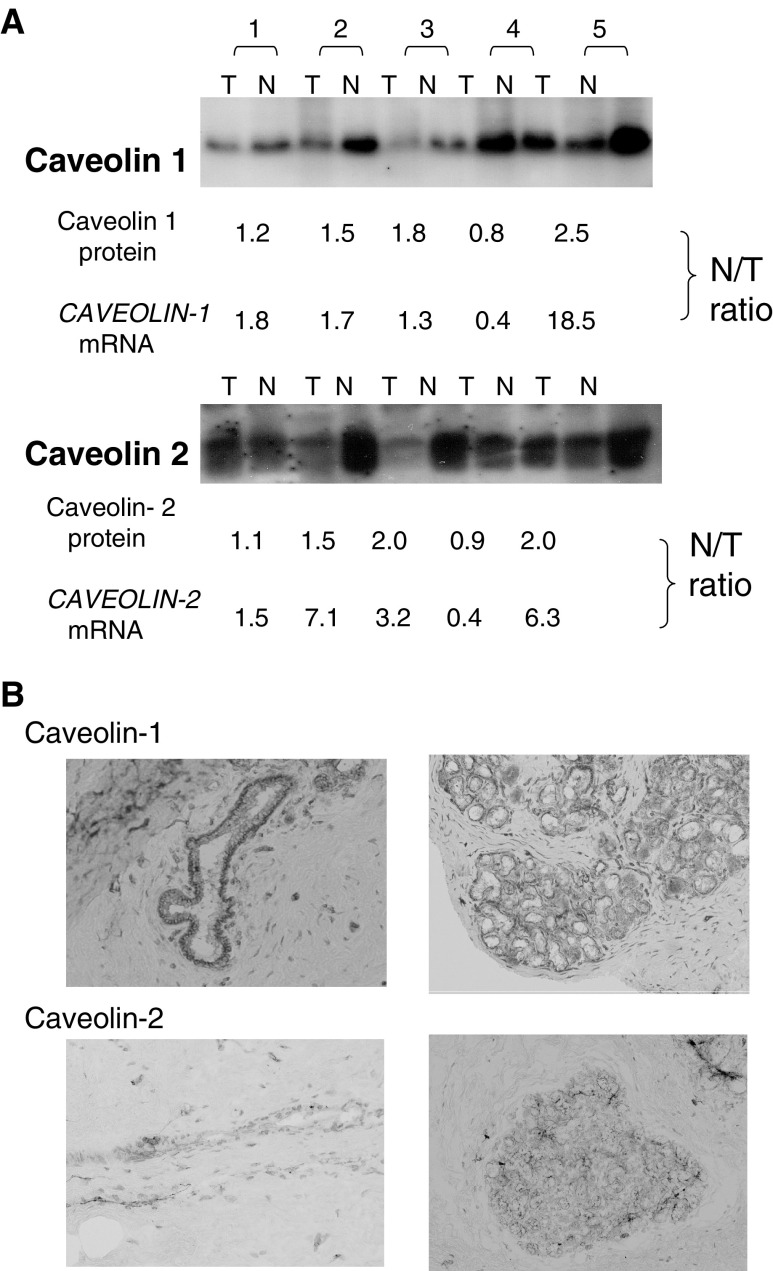
Western blotting and immunohistochemistry of CAV1, 2. (**A**) Protein expression of Caveolin-1 and Caveolin-2 in tumour and normal tissues from five breast cancer cases by Western blotting. Expression was measured using the free software program, NIH Image (version 1.62), and the expression ratio of normal to tumour (N/T) was calculated to compare with mRNA N/T ratios obtained by quantitative real-time RT–PCR. For instance, the N/T ratio in case #4 was the lowest at the protein level and at the mRNA level, while case #5 showed the highest N/T ratio for both protein and mRNA. Also, the N/T ratios for Caveolin-1 and Caveolin-2 in the representative five cases were concordant. (**B**) Localization of Caveolin-1 and Caveolin-2 expression by immunohistochemistry. Caveolin-1 (upper row) and Caveolin-2 (lower row) expression was abundant in mammary gland myoepithelial cells of (right) and in the mammary duct (left) in this representative normal breast tissue.

We preliminary confirmed the concordant expression between caveolins at the mRNA level by quantitative real-time PCR, and at protein level by Western blot analysis in five representative breast cancer samples (Figure 5

). CAV1 and CAV2 protein levels were downregulated in four representative breast cancer tissues compared to corresponding normal breast tissues.

Immunohistochemical assays showed that the expression of both caveolins was more abundant in the myoepithelial cells of the mammary duct than ductal epithelial cell in normal breast tissues, while fewer expressions of CAV1 and CAV2 proteins were detected in cancer tissues (data not shown).

### Clinicopathologic significance of HER2/neu and CAV1, 2

As shown in Table 2

**Table 2:** Relationship between clinicopathologic characteristics and expressions of caveolin (CAV)1, caveolin (CAV)2 and HER2

	**CAV1 expression**			**CAV2 expression**			**HER2 expression**		
	**Low**	**High**	***P*-value**	**Low**	**High**	***P*-value**	**Low**	**High**	***P*-value**
Age (years)			NS (0.530)			NS (0.753)			NS (0.116)
⩾55	39	42		39	41		35	45	
<55	42	39		42	41		46	36	
									
Size (cm)			0.041^a^			NS (0.083)			NS (0.875)
⩾2.0	44	31		43	32		37	38	
<2.0	37	50		38	49		44	43	
									
Menopaus			NS (0.512)			NS (0.743)			NS (0.326)
Pre	31	27		27	30		32	26	
Post	50	54		53	51		49	55	
									
*n*			NS (0.753)			NS (0.355)			NS (0.529)
Positive	43	40		42	36		41	37	
Negative	38	41		39	45		40	44	
									
Ly			NS (1.000)			NS (0.344)			NS (0.528)
Positive	37	37		40	34		35	39	
Negative	44	44		41	47		46	42	
									
V			NS (0.527)			NS (0.140)			NS (0.833)
Positive	12	15		17	10		13	14	
Negative	69	66		36	41		68	67	
									
ER			0.021^a^			NS (0.053)			0.029^a^
Positive	34	47		35	46		46	35	
Negative	28	16		27	17		16	28	
									
PgR			NS (0.081)			0.013^a^			0.019^a^
Positive	35	45		33	47		46	34	
Negative	27	18		29	16		16	29	

a Statistical significant differences were observed between ER or PgR status and Her2 expression, between size of tumours or ER status and CAV1 expression, between PgR status and CAV2 expression.

NS=not significant; n=lymph node metastasis; Ly=lymph vessel permiation; V=vascular vessel permiation.

, HER2/neu mRNA level showed an association with hormonal receptor status (HER2/ER, *P*=0.029:. HER2/PgR, *P*=0.019), and the high CAV1 mRNA level group tended to exhibit small tumour size (*P*=0.041) and positive ER status (*P*=0.021). Furthermore, there was a significant association between high CAV2 mRNA level and positive progesterone receptor status (*P*=0.013). However, there were no significant differences in gene expression with respect to disease-free survival (HER2, *P*=0.745: CAV1, *P*=0.520: CAV2, *P*=0.740). HER2/neu mRNA level was not associated with that of CAV1 or CAV2 mRNA levels in breast cancer samples (HER2/CAV1, *P*=0.758: HER2/CAV2, *P*=0.755) (Table 3

**Table 3:** Relationship among expressions of HER2, CAV1 and CAV2 in 162 cases of breast cancer^a^

	**CAV1 high**	**CAV1 low**	**Total**
HER2 high	42	39	81
HER2 low	39	42	81
Total	81	81	162
			*P*-value: NS^b^
			
	**CAV2 High**	**CAV2 low**	**Total**
HER2 high	40	41	00
HER2 low	41	40	00
Total	81	81	162
			*P*-value: NS^b^
			
	**CAV1 High**	**CAV1 Low**	**Total**
CAV2 high	59	22	00
CAV2 low	22	59	00
Total	81	81	162
			P^*^<0.001

a A significant association was observed between CAV1 and CAV2 expressions.

b NS=no significant difference.

). In contrast, CAV1 mRNA level was significantly associated with CAV2 mRNA level (*P*<0.001). There were no significant associations with respect to histopathologic classification in the 162 breast cancer cases.

## DISCUSSION

CAV1 has been reported to interact with various intracellular signalling pathways and is thought to suppress tumour growth in breast cancer cell lines (Lee *et al*, 1998; Razani *et al*, 2001; Fiucci *et al*, 2002). Prior studies have suggested that oncogenic transformation results in reduced cellular levels of caveolin (Glenney and Soppet, 1992; Sager *et al*, 1994; Koleske *et al*, 1995), and that this reduction probably contributes to a loss of caveolae (Koleske *et al*, 1995). Lee *et al* (1998) found that the CAV1 levels were inversely correlated to breast cancer progression *in vitro* and the overexpression of CAV1 resulted in substantial growth inhibition of breast tumour cells, which normally had no endogenous caveolin expression.

Our study confirmed that mRNA level of CAV1 and CAV2 were significantly downregulated in human breast cancer tissues compared to corresponding normal tissues (*P*<0.001), and that CAV1 and CAV2 mRNA levels were significantly correlated with each other in breast cancer cell lines and tissues. Hurlstone *et al* reported that CAV1 was expressed in the normal breast tissue by myoepithelial cells but not by ductal epithelial cells. This is in agreement with our present study that observed much more CAV1 and CAV2 expression in myoepithelial cells than in ductal epithelial cells in normal breast tissue by immunohistochemistry.

CAV1-null mice show a striking increase in the frequency and size of multifocal dysplastic lesions in mammary grands, with the nuclei and the nuclei of mammary grand cells showing anaplastic characteristics with increased mitotic figures (Williams *et al*, 2003). This suggests that CAV1-mediated interactions between myoepithelial cells and the rest of mammary gland may play an important role in oncogenesis. In addition, our study found that breast cancer patients with tumours that expressed low levels of CAV1 mRNA tended to have larger tumour sizes, which supported the hypothesis that CAV1 acts as a growth suppressor in breast tumours (Lee *et al*, 1998).

There was a significant correlation between high CAV1 and CAV2 mRNA level and positive hormonal receptor status. Razandi *et al* reported that E2 stimulates the synthesis CAV1 and CAV2 proteins and activated a CAV1 promoter/luciferase reporter construct transfected into in smooth muscle cells. CAV1 also stimulated ER translocation to the cell membrane in MCF-7 cells, inhibiting E2-induced ERK (MAPK) activation, required for DNA synthesis and cell survival (Razandi *et al*, 2002). Thus, our results support the hypothesis that CAV1 and CAV2 play an important role in the process of hormonal translocation.

Our present results also suggested that HER2/neu level and hormonal receptor expression was inversely correlated. This finding is supported by the Southwest Oncology Group Study and Konecney Report (Elledge *et al*, 1998; Ferrero-Pous *et al*, 2000; Konecny *et al*, 2003). While previous studies have reported HER2/neu to be a poor prognostic factor (Slamon *et al*, 1989; Quenel *et al*, 1995), neither CAV1, CAV2, nor HER2/neu appeared to be a predictor of disease outcome in our study. This may be due to the average follow-up period in our study being only 1141 days, such that few disease recurrences were observed. Thus, future studies may require longer observation periods and higher patient numbers for analysis.

Several mechanisms could be involved in the suppression of CAV gene expression observed in our study, including loss of heterozygosity (LOH) (Tatarelli *et al*, 2000; Zenklusen *et al*, 2001; Lee *et al*, 2002), point mutations (Hayashi *et al*, 2001) and methylation (Engelman *et al*, 1999). As for LOH, several LOH markers are located near the CAV1 gene locus on human chromosome 7q31. Loss of heterozygosity is frequently encountered in this region in a variety of human neoplasias, indicating the presence of a tumour-suppressor gene. With regard to mutation, Hayashi *et al* (2001) reported CAV1 gene mutations in approximately 16% of human breast cancers, and that these mutations may play a role in malignant progression. Although CAV1 expression is increased in prostate cancer (Yang *et al*, 1999), oesophageal cancer (Kato *et al*, 2002) and ovarian cancer (Davidson *et al*, 2001), future studies should include assessment of CAV1 mutation. As for methylation, Engelman *et al* (1999) reported that the first and second exons of the CAV1 and CAV2 genes are embedded within CpG islands, such that it is possible that caveolin gene expression is controlled, at least in part, by methylation of these CpG regions.

Zihui *et al* reported that growth factor receptor stimulation activated the phosphatidylinositol 3′-kinase and *β*-catenin pathways in mammary epithelial cells. *β*-Catenin activates cyclin D1 and c-Myc transcription, and subsequently, c-Myc suppressing caveolin-1 transcription (Xie *et al*, 2003). Furthermore, some studies have shown that activation of HER2/neu downregulates CAV1 expression *in vitro* (Couet *et al*, 1997; Kim *et al*, 2000), while other studies have reported the negative regulation of expression and signal transduction between CAV1 and HER2/neu (Engelman *et al*, 1998; Zhang *et al*, 2000). However, our present study revealed no such correlations *in vivo*. This may be due to a discrepancy between experimental and clinical studies.

In summery, our results indicated that CAV1 suppression correlated closely with that of CAV2 in breast cancer, that CAV1 level was inversely correlated with tumour size, and that CAV1 and CAV2 levels were correlated with hormonal receptor status. Therefore, CAV1 and CAV2 play an important role in tumour progression in breast cancer patients.
